# Improvement in the Thermal Stability of *Is*MHETase by Sequence and Structure-Guided Calculation

**DOI:** 10.3390/molecules30050988

**Published:** 2025-02-20

**Authors:** Shuyan Duan, Tianzhu Chao, Yaoyao Wu, Zhaoyi Wei, Sheng Cao

**Affiliations:** College of Food Science and Pharmaceutical Engineering, Zaozhuang University, Zaozhuang 277160, China

**Keywords:** *Is*MHETase, thermal stability, protein design

## Abstract

In the degradation of poly(ethylene terephthalate) (PET), mono(2-hydroxyethyl) terephthalate (MHET) hydrolase (*Is*MHETase) plays a crucial role in the complete degradation of PET. Although *Is*MHETase was discovered concurrently with *Is*PETase, its structural and functional properties are not well understood. To enhance the thermal stability of *Is*MHETase, we selected six homologous proteins that share the closest evolutionary relationship for structure-based protein rational design, all exhibiting over 60% amino acid sequence identity with *Is*MHETase. Using FireProt, PROSS, and Consensus analysis, we identified the key mutation sites of *Is*MHETase. Sequence and structural analyses indicate that, among these seven proteins, all amino acids within 5 Å of the substrate-binding site are identical, with the exception of Ser131 and Phe415. Additionally, the amino acids within a 4 Å range of the catalytic triad are nearly identical. Through integrated free energy calculations, phylogenetic tree analysis, sequence analysis, and conservation analysis, we have identified a variant with four key mutations (termed *Is*MHETase-M1: N156G, T159V, E110A, A493P) that exhibits improved thermal stability. The selection of mutations during the protein modification process often requires considerable time. Our predictions have established a foundation for the rational design of *Is*MHETase and its homologous proteins.

## 1. Introduction

Due to its physical and chemical properties, plastic plays an indispensable role in various aspects of human life [1,2]. However, as global production and consumption of plastic dramatically increase, plastic waste accumulates in the environment, posing a threat to both ecosystem and human health [1]. PET is one of the most widely used plastics in packaging and textile manufacturing [2].

In 2016, a new bacterial species, *Ideonella sakaiensis*, capable of utilizing PET as a carbon source, was isolated by Yoshida et al. [3]. This bacterium uses PET as its primary energy and carbon source through the action of two secreted enzymes: PET hydrolase (*Is*PETase) and mono(2-hydroxyethyl) terephthalate hydrolase (*Is*MHETase) [3]. *Is*PETase degrades PET to produce terephthalic acid (TPA), MHET, ethylene glycol (EG), and minor amounts of bis(2-hydroxyethyl) terephthalate (BHET) at moderate temperatures, exhibiting relatively higher activity than other PET-degrading enzymes, such as cutinases and lipases [4]. Subsequently, MHETase hydrolyzes MHET into TPA and EG [5]. Despite being discovered concurrently with *Is*PETase [3], *Is*MHETase, the enzyme essential for the complete degradation of PET, remains less understood.

Phylogenetic analysis has classified *Is*MHETase as a member of the tannase enzyme family, which includes fungal and bacterial tannases, as well as feruloyl esterases [6]. A few published studies have reported the structure of *Is*MHETase at resolutions ranging from 2.1 to 2.2 Å, highlighting its structural similarity to ferulic acid esterase (FEA) [6,7,8]. Recent studies have demonstrated that chimeric proteins, which covalently link the C-terminus of *Is*MHETase to the N-terminal end of *Is*PETase, exhibit enhanced turnover rates for PET and MHET compared to the free enzyme [7]. The hydrolysis of MHET by *Is*MHETase significantly mitigates the decline in the degradation activity of PET hydrolase caused by the accumulation of MHET in the reaction system. Furthermore, the extracellular production of *Is*MHETase functions as Exo-PETase by hydrolyzing the synthesized PET pentamer [6]. These findings suggest that *Is*MHETase plays a crucial supporting role in PET degradation, warranting further investigation.

Protein structure analysis provides valuable insights for modifying protein activity. Structure-guided mutagenesis of *Is*MHETase alters its substrate specificity towards MHET [6]. However, several questions regarding *Is*MHETase remain unanswered. For instance, its activity is relatively low and rapidly decreases when heated to 44 °C [8]. Therefore, research aimed at enhancing the thermal stability of *Is*MHETase is necessary.

Directed evolution and rational design are two primary methods for enhancing protein stability and catalytic activity [9]. Directed evolution involves constructing a large mutant library, and implementing this approach is labor-intensive [10]. Rational design relies on a comprehensive understanding of the relationship between protein structure and function [11]. Numerous types of studies have focused on single-point rational design based on protein structure. However, single-point mutations are usually predicted in silico and must be followed by laborious and costly protein expression, purification, and characterization [12]. Recently, three online servers, PROSS [13], FireProt [12], and Consensus Finder [14], used for the design of stable multiple-point mutants, have been reported. Despite the obvious need, these techniques have not yet been applied to the study of *Is*MHETase.

In this study, we combined three online servers to identify potential key sites for enhancing the thermal stability of *Is*MHETase. We selected six homologous proteins of *Is*MHETase that share a close evolutionary relationship for structure-based protein rational design, all exhibiting an amino acid sequence identity greater than 60% with *Is*MHETase. These proteins are tannase/feruloyl esterase family α/β hydrolases from *Pseudacidovorax* sp., *Comamonas thiooxydans*, *Hydrogenophaga* sp. *PML113*, an uncultured bacterium, *Caldilineaceae* bacterium, and *Burkholderiaceae* bacterium. Ultimately, we identified a variant termed *Is*MHETase-M1, which includes four key site mutations (N156G/T159V/E110A/A493P) with improved thermal stability.

## 2. Results

### 2.1. Sequence Analysis

**Figure 1:**
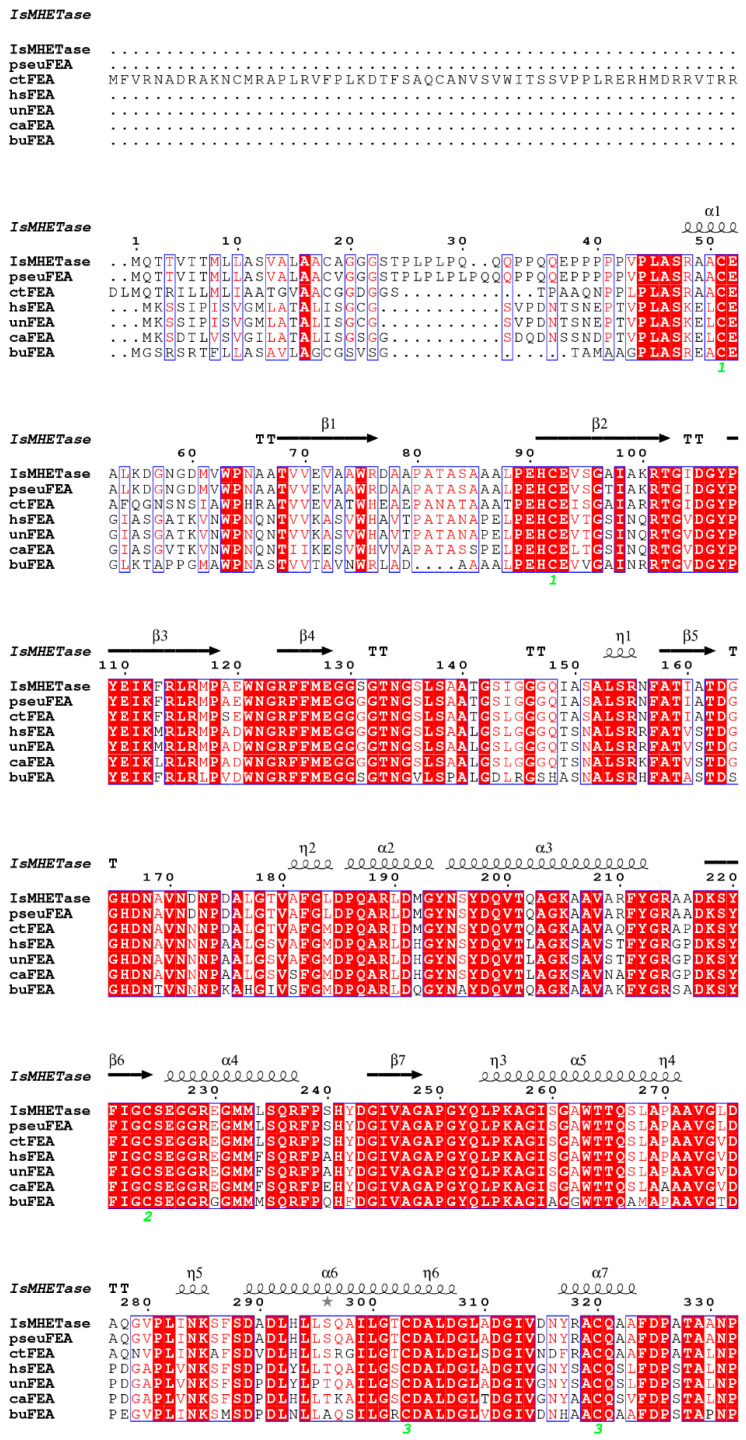

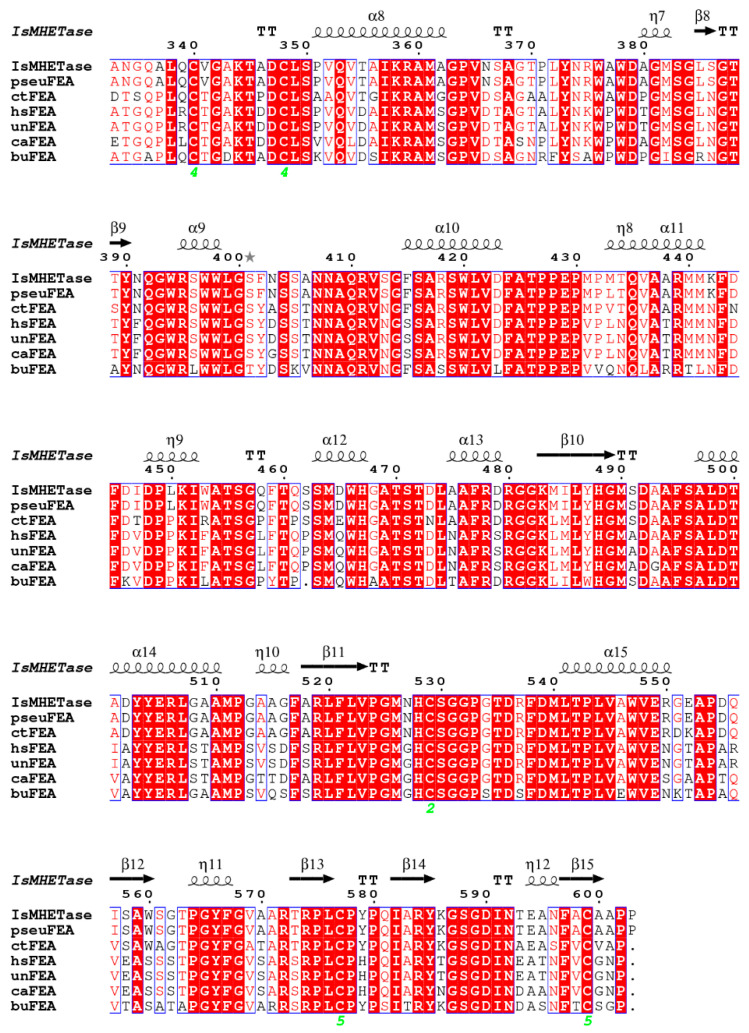
Sequence alignment of *Is*MHETase and its six homologous proteins from *Pseudacidovorax* sp. (pseuFEA), *Comamonas thiooxydans* (ctFEA), *Hydrogenophaga* sp. *PML113* (hsFEA), uncultured bacterium (unFEA), *Caldilineaceae* bacterium (caFEA), and *Burkholderiaceae* bacterium (buFEA), respectively. Secondary structure elements are drawn based on the *Is*MHETase structure. A red box with a white character, red character, and blue frame represents strict identity, similarity in a group, and similarity across groups, respectively. The cysteines that make up the 5 pairs of disulfide bonds are marked with green numbers.

To ensure the accuracy of the analysis, six homologous proteins of *Is*MHETase from the NCBI database, which share an amino acid sequence identity above 60% with *Is*MHETase, were selected. These proteins include tannase/feruloyl esterase from *Pseudacidovorax* sp. (termed pseuFEA), *Comamonas thiooxydans* (termed ctFEA), and *Hydrogenophaga* sp. *PML113* (termed hsFEA), an uncultured bacterium (termed unFEA), *Caldilineaceae* bacterium (termed caFEA), and *Burkholderiaceae* bacterium (termed buFEA), respectively (Figure 1). The theoretical molecular weights of all seven proteins are approximately 60 kDa (Table S1). The isoelectric points are primarily around 6, and the signal peptide types are predominantly of the Sec/SPII type (Table S2). For *Is*MHETase, the signal peptide sequence consists of the N-terminal 17 amino acids (MQTTVTTMLLASVALAA).

### 2.2. Homology Modeling

**Figure 2:**
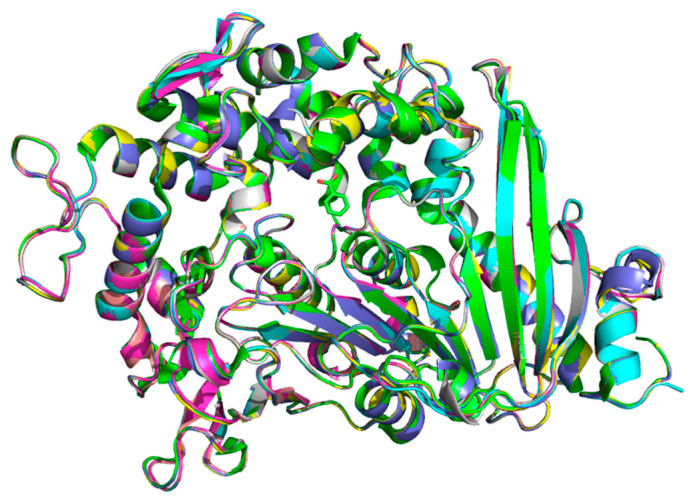
Structure alignment among *Is*MHETase and its six homologous proteins. *Is*MHET: green, unFEA: blue, pseu MHET: magenta, ctFEA: yellow, hsFEA: orange, buFEA: gray, caFEA: light blue.

The homology modeling results for all six proteins were highly plausible (Table S3). The structures of the six homologously modeled proteins closely resembled that of *Is*MHETase (Figure 2). The RMSD values in comparison to IsMHETase were all below 0.2 Å, with ctFEA demonstrating the greatest similarity, exhibiting an RMSD value of only 0.097 Å (Table S3).

### 2.3. Structure Alignment

Since the structures of *Is*MHETase and its six homologous proteins are highly similar, and there are no substrates present in the structural models, we used the substrate of *Is*MHETase to analyze the functional regions of all seven proteins. In previous work, Brandon C. Knott et al. identified the structure of *Is*MHETase [7]. Here, structural and sequence analyses of *Is*MHETase and its six homologs revealed that the gate residue F415 and the key residues involved in enzyme catalysis and substrate binding are nearly identical (Table S4). Except for Ser131 and Phe415, the amino acids within a 5 Å distance from the substrate binding site are identical (Table S5). Additionally, the amino acids within a 4 Å range of the catalytic triad are also nearly identical (Table S7).

**Figure 3:**
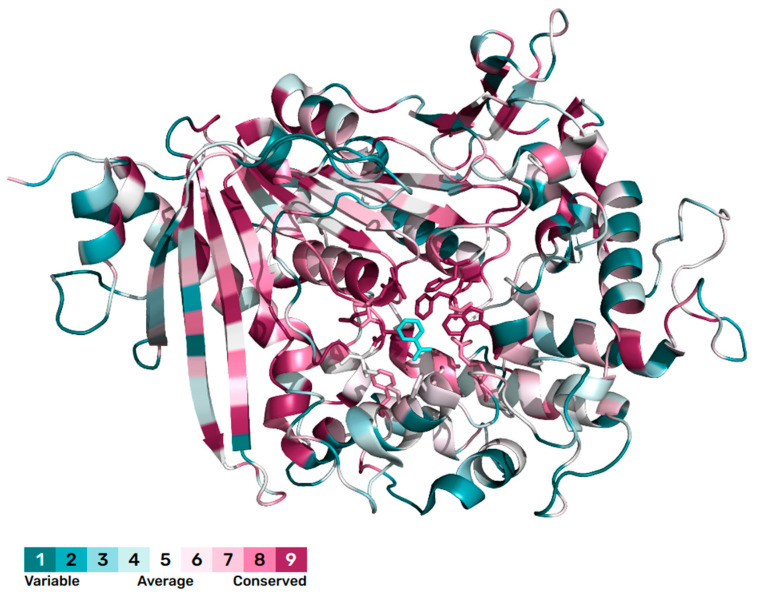
Consurf analysis of *Is*MHETase. The substrate is shown as sticks (blue). The amino acids that are within 5 Å of the substrate are shown as sticks.

**Figure 4:**
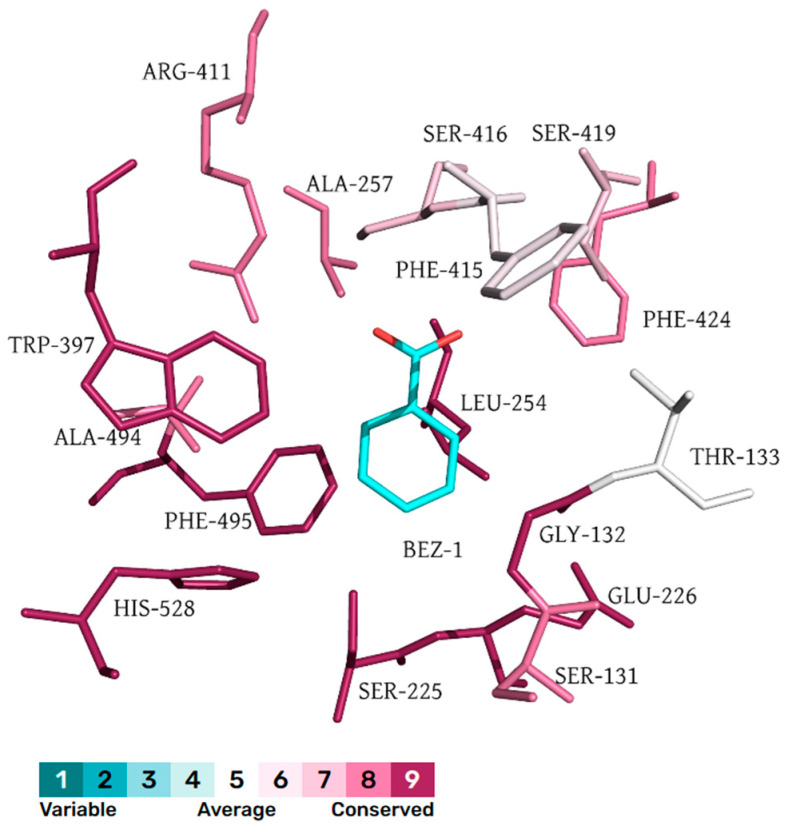
The amino acids that are within 5 Å of the substrate of *Is*MHETase.

The sequence conservation was analyzed using the ConSurf Server [15] to study the evolutionary conservation of the above-mentioned key amino acids. The calculation is performed on 150 sequences that are homologous to *Is*MHETase. For better visualization, the conservation scores are divided into nine grades by the ConSurf Server, the most variable amino acids grading 1 (cerulean), the intermediately conserved amino acids grading 5 (white), and the most conserved amino acids grading 9 (scarlet). Conservation analysis results indicate that the substrate binding region is highly conserved, while the protein surface region exhibits greater variability (Figure 3). Among the amino acids located within 5 Å of the substrate binding site, T133 and F415 are classified as intermediately conserved, whereas the remaining amino acids are highly conserved, especially W397, H528, S225, and F495 (Figure 4).

### 2.4. Identification of Mutation Sites

FireProt (https://loschmidt.chemi.muni.cz/fireprotweb/, accessed on 22 January 2025) enables users to directly analyze and optionally modify designed thermostable mutants. The results of the combination design include mutations derived from the energy-based approach as well as those suggested by the evolution-based approach [12]. PROSS is based on the automated design of protein structure and sequences to enhance bacterial expression and stability [13]. Generally, conserved amino acids are more likely to contribute to the stability of protein folding than non-conserved amino acids. Replacing a rarely occurring amino acid in a target protein with a frequently occurring amino acid from a homologous sequence is likely to increase its stability. Consensus Finder is based on this principle for mutation site prediction [14]. There are typical examples of successful protein designs utilizing these three methods. Consequently, we conducted a mutational analysis of IsMHETase using the three aforementioned methods. The PROSS prediction results revealed multiple mutation combinations (Table S8). To ensure a valid analysis, we selected the design4 mutation combination from PROSS based on the number of mutations predicted by FireProt (Table S9). FireProt predicted 58 mutant loci, while PROSS design4 predicted 56 mutant loci. By applying screening criteria that focused on amino acid sites differing from 75% of homologous proteins, Consensus Finder identified 13 amino acid mutation sites. Among these, N156G, T159V, E110A, and A493P were the four mutation sites identified by all methods (Table S6). Consequently, we constructed a multi-mutant incorporating these four mutation sites, designated *Is*MHETase-M1.

### 2.5. Expression and Purification of IsMHETase and IsMHETase-M1

**Figure 5:**
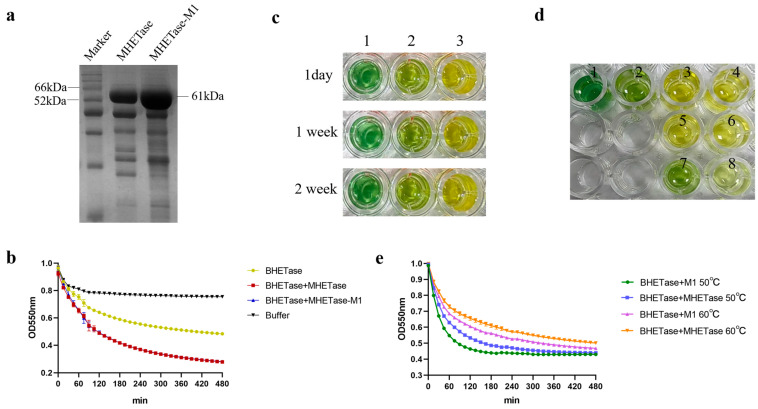
(**a**) SDS-PAGE analysis of *Is*MHETase and *Is*MHETase-M1. (**b**) Colorimetric activity assay of MHETase at 550 nm (for phenol red) was measured every 15 min for 8 h. (**c**) Color change colorimetric activity assay. 1: without BHETase and *Is*MHETase; 2: only BHETase; 3: BHETase and *Is*MHETase. (**d**) Thermal stability assay. 1: without enzyme; 2: only BHETase; 3: BHETase and *Is*MHETase; 4: BHETase and *Is*MHETase-M1; 5: BHETase and *Is*MHETase (50 °C, 15 min); 6: BHETase and *Is*MHETase-M1 (50 °C, 15 min); 7: BHETase and *Is*MHETase (60 °C, 15 min); 8: BHETase and *Is*MHETase-M1 (60 °C, 15 min). (**e**) Thermal stability of MHETase using colorimetric activity assay.

*Is*MHETase and *Is*MHETase-M1 were expressed as soluble proteins in *E. coli* Shuffle T7 cells. After purification using Ni affinity chromatography, the target proteins were obtained with high purity. The molecular weights of the two proteins were identical, approximately 61 kDa (Figure 5a).

### 2.6. Colorimetric Activity Assay for Determining the Optimal Reaction Concentrations of BHETase and MHETase

**Figure 6:**
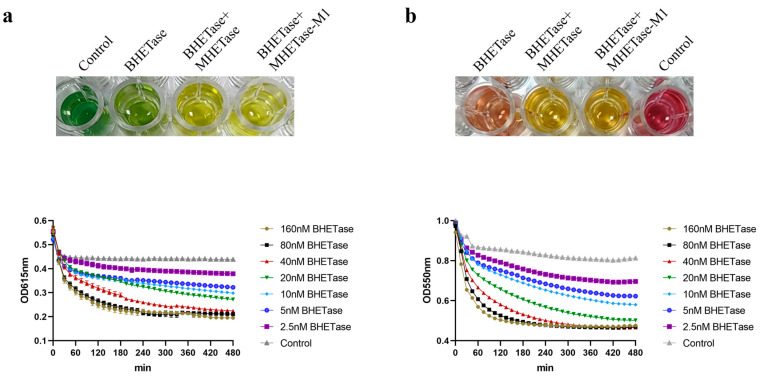
(**a**) Colorimetric activity assay of different concentrations of BHETase at 615 nm, color change in the upper part of the figure. (**b**) Colorimetric activity assay of different concentrations of BHETase at 550 nm, color change in the upper part of the figure.

In 2022, Jessica Lusty Beech et al. reported a method for determining whether a protein functions as a BHETase based on the color reaction of phenol red or bromophenol blue [16]. This colorimetric activity assay method offers a convenient and cost-effective assay for laboratories that lack access to high-performance liquid chromatography (HPLC), in contrast to commonly used techniques that necessitate HPLC for activity determination. Here, we first hydrolyzed BHET with a BHETase in our laboratory, which has BHET hydrolase activity but with limited MHET hydrolase activity. Then, we set up a control with *Is*MHETase added to this system and recorded the color change to determine whether this method could be used to assay MHETase of MHET hydrolysis activity. The experimental results showed that for bromophenol blue after a 1-day completion reaction, the reaction system of the experimental group to which only BHETase was added changed to a yellow-green color compared to the green color of the reaction system of the negative control group, while the reaction system of the experimental group to which both BHETase and *Is*MHETase were added changed to a yellow color (Figure 5c). Meanwhile, to ensure the accuracy of the color reaction, we monitored the color of this reaction system continuously for 2 weeks (Figure 5c), and the above results showed that this method is simple and reliable for the confirmation and comparison of MHETase hydrolase activity. A similar effect was observed in the phenol red assay, which appeared purplish-red in the negative control group, orange-red in the BHETase-only group, and yellow in the group that included both BHETase and MHETase (Figure 6b).

First, the visible/UV absorbance at 615 nm (for bromophenol blue) and 550 nm (for phenol red) was measured every 15 min for 8 h at 37 °C to determine the optimal concentrations of BHETase required for activity assays. The assay results indicated that a 40 nM BHETase reaction over 8 h produced effects comparable to those observed with 80 nM BHETase or 160 nM BHETase. Furthermore, the trends in absorbance readings at 550 nm and 615 nm were consistent; however, the effects were more pronounced at 550 nm wavelength (Figure 6a,b). The method for choosing the concentration of MHETase required for the reaction is consistent with the procedure for determining the concentration of BHETase. Ultimately, an ideal protein concentration of 100 nM MHETase was chosen for the activity detection experiment (Figure S1). Consequently, we selected the condition of 40 nM BHETase and 100 nM BHETase at 550 nm for the subsequent MHETase activity assay. The results demonstrated that the absorbance changes at 550 nm following the addition of MHETase or MHETase-M1 were nearly identical, indicating that MHETase and MHETase-M1 exhibited the same level of activity (Figure 5b).

### 2.7. IsMHETase-M1 with Enhanced Thermal Stability

Having established that the color reaction can be used to test for MHET hydrolysis activity in proteins, we employed this method to compare the thermal stability of *Is*MHETase and *Is*MHETase-M1. Compared to the untreated control, the reaction system turned yellow after an overnight reaction of *Is*MHETase and *Is*MHETase-M1 at 50 °C for 15 min, with no significant difference compared to the reaction system, indicating that it could still catalyze the complete hydrolysis of MHET (Figure 5d). The reaction system of *Is*MHETase, after heat treatment at 60 °C for 15 min, appeared yellowish-green, which was nearly identical to the color of the control group without *Is*MHETase. In contrast, the reaction system of *Is*MHETase-M1 after heat treatment at 60 °C for 15 min was yellowish (Figure 5d), indicating that some MHET had been hydrolyzed. The result of the colorimetric analysis is consistent with the result of the color reaction (Figure 5e). These results suggest that *Is*MHETase-M1 exhibits improved thermal stability compared to *Is*MHETase.

## 3. Discussion

Currently, only a limited number of published studies have focused on the structural and functional analysis of *Is*MHETase. Notably, *Is*MHETase exhibits structural similarities to ferulic acid esterase [4]. *Is*MHETase showed very weak activity against *p*NP-aliphatic esters, or typical aromatic ester compounds that can be catalyzed by enzymes from the tannase family [3]. Phylogenetic analysis has classified MHETase within the tannase enzyme family, which encompasses fungal and bacterial tannases as well as feruloyl esterases [6]. The question of whether MHETase is derived from a tannase or a feruloyl esterase ancestor remains unanswered [6]. Investigating its homologous proteins will enhance our understanding of this protein family and provide insights for engineering bifunctional enzymes.

Therefore, we predicted key mutation sites for *Is*MHETase and its six highly homologous proteins. The pI of ctFEA and buFEA differ significantly from those of the other five proteins. Specifically, the pI of ctFEA is 6.79 and the pI of buFEA is 7.55, while the pI of the other five proteins is approximately 5.0. Indeed, a near-neutral pI is often associated with poor solubility, and surface “supercharging” has been employed by others to enhance thermal stability [17]. This suggests that rational designing of ctFEA and buFEA to achieve an isoelectric point of around 5.0 may be an effective strategy.

**Figure 7:**
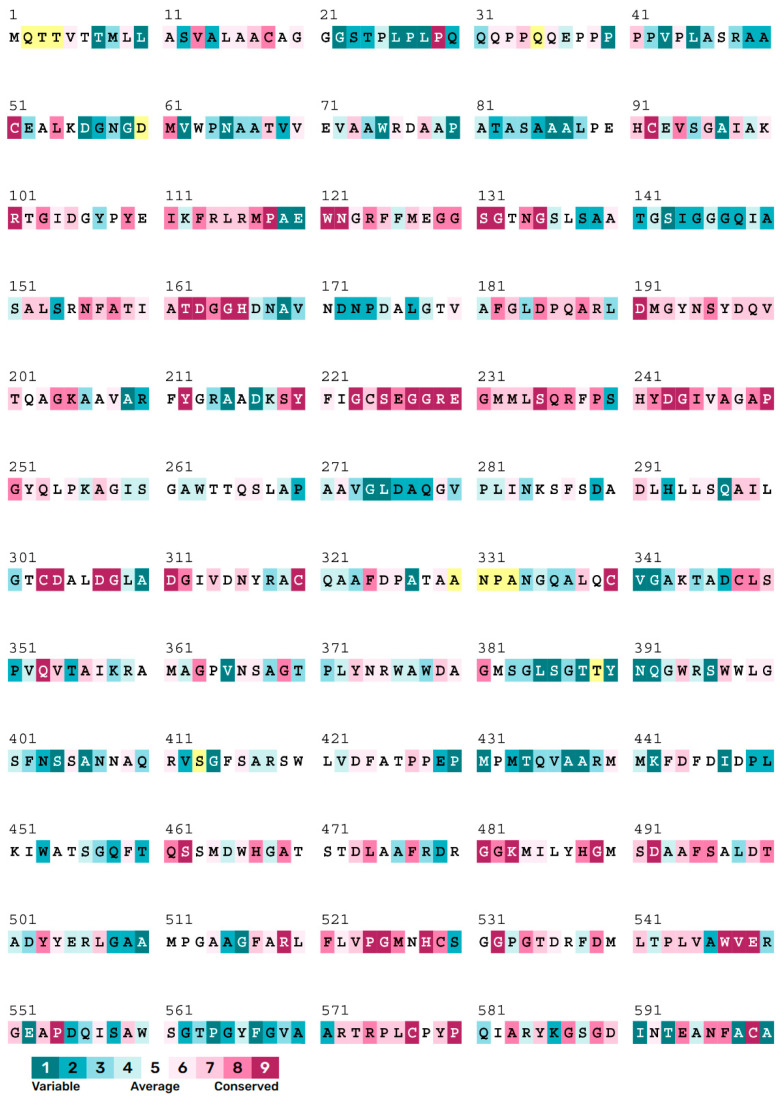
Consurf score of *Is*MHETase. The yellow boxes in the diagram represent that the calculation for this site was performed on less than 10% of the sequences.

Evolutionary analysis indicates a relatively low conservation of the gate residue F415 (Figure 7). The FireProt prediction results demonstrate that the F415M mutation leads to a decrease in free energy, which is considered a beneficial mutation. Previous studies have shown that the mutation of F415 to serine significantly reduces the hydrolytic activity of the *Is*MHETase enzyme on MHET [6]. However, there is currently no research investigating whether this mutation affects the enzyme’s activity as a ferulic acid esterase. This amino acid may represent a critical site for engineering the bifunctional enzyme of *Is*MHETase.

Furthermore, polyethylene furanoate (PEF) is a suitable alternative to PET in bottle production [4]. This innovative plastic PEF can be degraded by *Is*PETase [4]. The resulting product hydroxyethyl-2,5-furan dicarboxylate (referred to as MHEF) is sufficiently similar to MHET. Therefore, structure-guided mutagenesis of *Is*MHETase is likely to evolve into an enzyme termed “MHEFase”.

## 4. Materials and Methods

### 4.1. Materials

The coding sequence of *Is*MHETase (amino acids 18–603, excluding the signal peptide) was codon-optimized and subcloned into the pColdII expression plasmid, which contains an N-terminal 6xHis tag. The gene was synthesized by Sangon (Shanghai, China). The coding sequence of *Is*MHETase-M1 was mutated based on the *Is*MHETase sequence and was also obtained through gene synthesis at Sangon (Shanghai, China). SHuffle T7 *E. coli* competent cells were purchased from WEIDI Biotech (Shanghai, China). BHET was obtained from Sigma (Saint Louis, MO, USA), and BHET hydrolase is stored in our laboratory. Phenol red and bromophenol blue reagents were purchased from Macklin (Shanghai, China). Other general reagents were purchased from Beyotime (Shanghai, China).

### 4.2. Sequence Analysis

Six homologous proteins with more than 60% sequence similarity with *Is*MHETase were obtained from the NCBI blast database (Available online: https://blast.ncbi.nlm.nih.gov/Blast.cgi, accessed on 22 January 2025) using the amino acid sequence of *Is*MHETase (UniProt: A0A0K8P8E7) as the search template. Sequence alignment was conducted using the CLUSTALW server (Available online: www.genome.jp/tools-bin/clustalw, accessed on 22 January 2025) [18]. Following this, the multiple sequence alignment was enhanced visually using ESPript 3.0 (Available online: https://espript.ibcp.fr/ESPript/ESPript/, accessed on 22 January 2025) [19]. The presence of signal peptides and the identification of their cleavage sites in proteins were predicted using the SignalP 5.0 server [20]. The theoretical isoelectric point (pI) and molecular weight (MW) of proteins were computed in Expasy (Available online: https://www.expasy.org/, accessed on 22 January 2025) [21].

### 4.3. Homology Modeling

The homology models of the proteins were generated using the Swiss Model (Available online: https://swissmodel.expasy.org/, accessed on 22 January 2025) [22] through multiple target modes. Homology models for all six homologous proteins were constructed using *Is*MHETase (PDB: 6QZ3) as the search template. The quality of the modeled structures was validated using the “Structure Assessment” module in the Swiss-Model server. All structural alignments and analyses were performed using PyMOL (https://www.pymol.org/, accessed on 22 January 2025). The evolutionary conservation profiles of the protein structures were predicted using the ConSurf server (Available online: https://consurf.tau.ac.il/, accessed on 22 January 2025) [15].

### 4.4. FireProt Design

The FireProt tool integrates energy-based and evolution-based approaches to design reliable and stable multiple-point mutants [12]. The required input for the FireProt web server is the tertiary structure of the protein of interest, which can be provided either as a PDB ID or as a user-defined PDB file.

### 4.5. PROSS

PROSS (Available online: https://pross.weizmann.ac.il/step/pross-terms/, accessed on 22 January 2025) utilizes Rosetta modeling and phylogenetic sequence information in its computational core [13]. The required input file for the PROSS web server is the tertiary structure of the user-defined PDB file, along with the corresponding amino acid sequence or a provided PDB ID.

### 4.6. Consensus Finder

Typically, mutating a protein to resemble the consensus sequence of its homolog increases its thermal stability and enhances soluble expression during recombinant production [14]. Consensus Finder (Available online: http://kazlab.umn.edu/, accessed on 22 January 2025) identifies the consensus sequence of the target to the homolog and predicts potentially stable mutations. The application requires either the PDB code or a FASTA file of the protein.

### 4.7. Protein Expression and Purification

The protein expression of *Is*MHETase and *Is*MHETase-M1, with the signal peptide (amino acids 1–17) removed, was conducted. For clarity, we have retained the original numbering of the amino acids in the text. The methodology is as follows. The corresponding plasmids were transformed into *E. coli* Shuffle T7 expression cells and screened on LB solid agar plates containing 100 mg/L ampicillin. After overnight incubation at 37 °C, a single colony from the solid medium was selected and transferred to 50 mL of LB liquid medium with the same concentration of ampicillin. Subsequently, 20 mL of this bacterial culture was added to 1 L of LB liquid medium containing 100 mg/L ampicillin. The cells were incubated at 37 °C with shaking at 220 rpm until the optical density (OD) at 600 nm reached 0.6. The temperature was then lowered to 18 °C, and 0.3 mM isopropyl β-D-1-thiogalactopyranoside was added to the cells to induce protein expression.

Cells were collected through centrifugation and subsequently lysed using a bacterial lysate. The supernatant was collected after centrifugation at 10,000 rpm. This supernatant was then added to a nickel affinity chromatography medium that had been equilibrated with an equilibrium solution consisting of 50 mM Tris-HCl (pH 8.0), 500 mM NaCl, 10 mM imidazole, and 5% glycerol. After loading the samples, they were subjected to a gradient elution with eluents containing 10 mM, 50 mM, and 250 mM imidazole, respectively. Finally, after analyzing protein purity using 12.5% SDS-PAGE, the proteins were concentrated, the buffer was changed to 20 mM Tris, and 100 mM NaCl, and finally, the proteins were frozen at −80 °C for subsequent experiments.

### 4.8. Colorimetric Activity Assay

The bromophenol blue color reaction assay was optimized based on the work of Jessica Lusty Beech et al. (Figure S2) [16]. The reaction system was established as follows: 40 nM BHETase, 1 mM BHET, 0.1 mM bromophenol blue or 0.1 mM phenol red, 10 mM CaCl_2_, 10% DMSO, 5 mM BES (pH 7.0) or 5 mM HEPES (pH 8.0) per 200 μL, and 100 nM *Is*MHETase or *Is*MHETase-M1 for the reaction group, with an equal volume of protein storage buffer (20 mM Tris, and 100 mM NaCl) for the control group. The total volume of the reaction system was replenished to 200 μL with distilled water, and a negative control without any enzyme was set up. The above reaction system was transferred to a 96-well plate and left at room temperature overnight to observe the color change. Visible/UV absorbance at 615 nm (for bromophenol blue) or 550 nm (for phenol red) was measured every 15 min for 8 h at 37 °C using a plate reader.

### 4.9. Thermal Stability Assay

The reaction system for the thermal stability assay was established as follows; 40 nM BHETase, 1 mM BHET, 0.1 mM bromophenol blue, 10 mM CaCl_2_, 10% DMSO, and 5 mM BES (pH 7.0) were added to each 200 μL of the reaction system. The reaction groups were added with 100 nM *Is*MHETase or *Is*MHETase-M1, which were subjected to different heat treatments: heat-treated, heat-treated for 15 min at 50 °C, and heat-treated for 15 min at 60 °C, respectively. The control group was added with an equal volume of protein storage buffer. Additionally, a negative control group without any enzyme was included. The above reaction system was transferred to a 96-well plate and left incubated at room temperature overnight to monitor the color change. Visible/UV absorbance of the heat-treated groups at 615 nm (for bromophenol blue) or 550 nm (for phenol red) was measured every 15 min for 8 h at 37 °C using a plate reader.

## 5. Conclusions

The selection of mutations in the protein modification process can be time-consuming. By integrating free energy calculations, phylogenetic tree analysis, sequence analysis, and conservation analysis, we identified a mutation, *Is*MHETase-M1, that enhances stability. This mutation has been tested for activity and thermal stability before and after the modification, utilizing a straightforward detection method. We thus anticipate that our structural characterization and rational design of *Is*MHETase provide ideas for the design of MHETase and MHEFase.

## Data Availability

The data supporting the findings of this study can be obtained from the corresponding author upon reasonable request.

## Supplementary Materials

The following supporting information can be downloaded at: https://www.mdpi.com/article/10.3390/molecules30050988/s1, Figure S1: Colorimetric activity assay of different concentrations of MHETase at 550 nm; Figure S2: Schematic diagram of BHET degradation by BHETase and MHETase. The color change associated with the indicator is illustrated in the figure; Table S1. Protein pI and MW Analysis of *Is*MHETase and Its Six Homologous Proteins; Table S2. Signal peptide analysis of *Is*MHETase and Its Six Homologous Proteins; Table S3. Structure Alignment of *Is*MHETase and Its Six Homologous Proteins; Table S4. Key Amino Acids in Catalytic and Substrate Binding of *Is*MHETase and Its Six Homologous Proteins; Table S5. Amino Acids in the 5 Å Range from the Substrate of *Is*MHETase and Its Six Homologous Proteins; Table S6. The combination of mutation sites for *Is*MHETase predicted by the three software programs; Table S7. Amino acids in the 4 Å range from the catalytic triplex; Table S8. Key Amino Acids of *Is*MHETase Predicted Using PROSS; Table S9. Key Amino Acids of *Is*MHETase Predicted Using FireProt.
