# Supplementary material for: Improvement in the Thermal Stability of IsMHETase by Sequence and Structure-Guided Calculation
Source: Molecules. 2025 Feb 20;30(5):988. doi: 10.3390/molecules30050988 (PMC11902034; doi:10.3390/molecules30050988)
Supplement: Supplementary file 1 [file molecules-30-00988-s001.zip › molecules-3468299-supplementary.pdf]

Supplementary Materials

Improvement in the Thermal Stability of *Is*MHETase by Sequence and Structure-Guided Calculation

Table S1. Protein pI and MW Analysis of *Is*MHETase and Its Six Homologous Proteins.

Protein	Theoretical pI	Theoretical MW
*Is*MHETase	5.18	63102.86
pseuFEA	5.18	63335.21
ctFEA	6.79	68937.47
hsFEA	5.31	62346.63
unFEA	5.31	62330.59
caFEA	5.03	62362.44
buFEA	7.55	61574.17

Table S2. Signal peptide analysis of *Is*MHETase and Its Six Homologous Proteins.

Protein	Signal peptide (Sec/SPI)	Signal peptide (Tat/SPI)	Signal peptide (Sec/SPII)	Other
*Is*MHETase			√	
pseuFEA			√	
ctFEA				√
hsFEA			√	
unFEA			√	
caFEA			√	
buFEA		√		

Table S3. Structure Alignment of *Is*MHETase and Its Six Homologous Proteins.

Protein	Ramachandran Favoured	Ramachandran Outliers	QMEANDisCo Global	MolProbity Score	RMSD
*Is*MHETase	-	-	-	-	-
pseuFEA	95.89%	0.36%	0.95 ± 0.05	0.88	0.184Å（478 to 478 atoms）
ctFEA	96.07%	0.36%	0.92 ± 0.05	1.06	0.097Å（528 to 528 atoms）
hsFEA	96.60%	0.36%	0.91 ± 0.05	1.04	0.186Å（400 to 400 atoms）
unFEA	96.42%	0.36%	0.91 ± 0.05	1.00	0.198Å（489 to 489 atoms）
caFEA	96.61%	0.18%	0.91 ± 0.05	1.1	0.099Å（524 to 524 atoms）
buFEA	94.77%	0.72%	0.89 ± 0.05	1.23	0.195Å（487 to 487 atoms）

Table S4. Key Amino Acids in Catalytic and Substrate Binding of *Is*MHETase and Its Six Homologous Proteins.

Protein	Enzyme catalytic			Substrate binding				Gate residue
	225	492	528	254	397	411	416	415
*Is*MHETase	S	D	H	L	W	R	S	F
PseuFEA	S	D	H	L	W	R	S	F
ctFEA	S	D	H	L	W	R	S	F
hsFEA	S	D	H	L	W	R	S	S
unFEA	S	D	H	L	W	R	S	S
caFEA	S	D	H	L	W	R	S	S
buFEA	S	D	H	L	W	R	S	F

Table S5. Amino Acids in the 5 Å Range from the Substrate of *Is*MHETase and Its Six Homologous Proteins.

Protein	Substrate distance (<5Å)															
	131	132	133	225	226	254	257	397	411	415	416	419	424	494	495	528
*Is*MHETase	S	G	T	S	E	L	A	W	R	F	S	S	F	A	F	H
pseuFEA	G	G	T	S	E	L	A	W	R	F	S	S	F	A	F	H
ctFEA	G	G	T	S	E	L	A	W	R	F	S	S	F	A	F	H
hsFEA	G	G	T	S	E	L	A	W	R	S	S	S	F	A	F	H
unFEA	G	G	T	S	E	L	A	W	R	S	S	S	F	A	F	H
caFEA	G	G	T	S	E	L	A	W	R	S	S	S	F	A	F	H
buFEA	S	G	T	S	E	L	A	W	R	S	S	S	F	A	F	H

Table S6. The combination of mutation sites for *Is*MHETase predicted by the three software programs.

PROSS	FireProt	Consensus finder	Mutation sites
E110A	E110A	E110A	E110A
	M117L	M117L	
S131G		S131G	
N156G	N156G	N156G	N156G
T159V	T159V	T159V	T159V
A493P	A493P	A493P	A493P
G534A		G534A	
E594A		E594A	

Table S7. Amino acids in the 4Å range from the catalytic triplex.

Protein	Catalytic triplex distance (<4 Å)																								
	224	225	226	227	228	229	248	249	251	250	376	397	488	489	490	491	492	493	494	495	526	527	528	529	530
*Is*MHETase	C	S	E	G	G	R	G	A	G	P	W	W	H	G	M	S	D	A	A	F	M	N	H	C	S
pseuFEA	C	S	E	G	G	R	G	A	G	P	W	W	H	G	M	S	D	A	A	F	M	N	H	C	S
ctFEA	C	S	E	G	G	R	G	A	G	P	W	W	H	G	M	S	D	A	A	F	M	N	H	C	S
hsFEA	C	S	E	G	G	R	G	A	G	P	W	W	H	G	M	A	D	A	A	F	M	G	H	C	S
unFEA	C	S	E	G	G	R	G	A	G	P	W	W	H	G	M	S	D	G	A	F	M	N	H	C	S
caFEA	C	S	E	G	G	R	G	A	G	P	W	W	H	G	M	A	D	A	A	F	M	G	H	C	S
buFEA	C	S	E	G	G	R	G	A	G	P	W	W	H	G	M	S	D	A	A	F	M	G	H	C	S

Table S8. Key Amino Acids of IsMHETase Predicted Using PROSS.

Wild type	design1	design2	design3	design4
A150				T
A330				L
A78				P
F402				Y
L234				M
S131				G
S296				A
S491				A
W453				Y
Y252				F
A87			P	P
D172			N	N
G516			D	D
H293			K	K
L268			F	F
T563			N	N
A493		P	P	P
A81		P	P	P
E594		A	A	A
G258		A	A	A
G261		A	A	A
H467		V	V	V
L190		I	I	I
L450		A	Q	Q
M192		W	W	F
N156		G	G	G
Q458		P	P	P
R439		F	Y	Y
S267		A	A	A
S286		A	A	A
S416		V	V	V
T141		L	L	L
V208		I	I	I
V70		I	I	I
Y107		K	Q	Q
A216	P	P	P	P
A323	V	V	V	V
A337	P	P	P	P
A79	V	V	V	V
A99	N	N	N	N
E110	A	A	A	A
E226	N	T	T	T
G301	A	A	A	A
G508	E	E	E	E
G534	A	A	A	A
H241	Y	Y	Y	Y
I447	R	R	R	R
K218	R	R	R	R
N284	Q	Q	Q	Q
P449	Y	Y	Y	Y
P543	A	A	A	A
S260	A	A	A	A
S95	T	T	T	T
T133	L	L	L	L
T159	V	V	V	V
T593	D	D	D	D

Table S9. Key Amino Acids of IsMHETase Predicted Using FireProt.

Chain	Mutation	Conserved	Fold X [kcal/mol]	Rosetta [kcal/mol]
A	A79F	N	-1.21	-8.14
A	A85L	N	-1.42	-2.46
A	I98M	N	0.31	-
A	A99N	N	-1.11	-3.78
A	E110A	N	-1.26	-1.59
A	M117L	Y	0.09	-
A	S151Y	N	-2.25	-3.37
A	N156G	Y	-0.35	-
A	T159V	Y	-0.10	-
A	A161S	Y	0.01	-
A	T179P	N	-2.12	-8.77
A	L190Y	N	-1.26	-2.45
A	M192Y	Y	-0.74	-
A	S196A	Y	-0.13	-
A	G204A	Y	0.18	-
A	A209M	N	-1.00	-3.59
A	A216P	Y	-1.95	-
A	K218R	Y	-0.15	-
A	E226N	Y	-0.36	-
A	G231A	Y	-0.43	-
A	S235A	N	-0.70	-
A	S240W	N	-1.05	-2.72
A	Y252F	Y	-0.30	-
A	S260A	N	-0.64	-
A	T264V	N	-2.33	-2.32
A	L268F	N	-1.26	-5.47
A	I283L	N	0.11	-
A	S286A	N	-0.82	-
A	G301A	N	-0.97	-
A	T302L	N	-1.37	-3.09
A	A305Y	N	-1.08	-3.48
A	N316D	N	-0.07	-
A	A322F	N	-0.51	-
A	A337P	N	-1.12	-2.46
A	A346G	N	-0.20	-
A	V352A	N	0.08	-
A	I357L	N	0.43	-
A	V365Y	N	-1.01	-2.31
A	A377P	N	-2.10	-1.27
A	F415M	N	-1.47	-4.69
A	S419A	N	-0.08	-
A	L450P	N	-1.13	24.07
A	F459Y	Y	-0.65	-
A	Q461E	N	0.34	-
A	H467M	N	-1.96	0.36
A	T470P	N	-0.67	-
A	M484L	Y	0.22	-
A	A493P	Y	-1.97	-
A	A494V	N	0.27	-
A	A497P	N	-2.48	-3.45
A	Y503W	Y	-0.54	-
A	G508Y	N	-1.14	-6.05
A	A510F	N	-1.07	-2.45
A	A546L	N	-1.04	-2.96
A	S561A	N	-0.62	-
A	G565A	N	-0.21	-

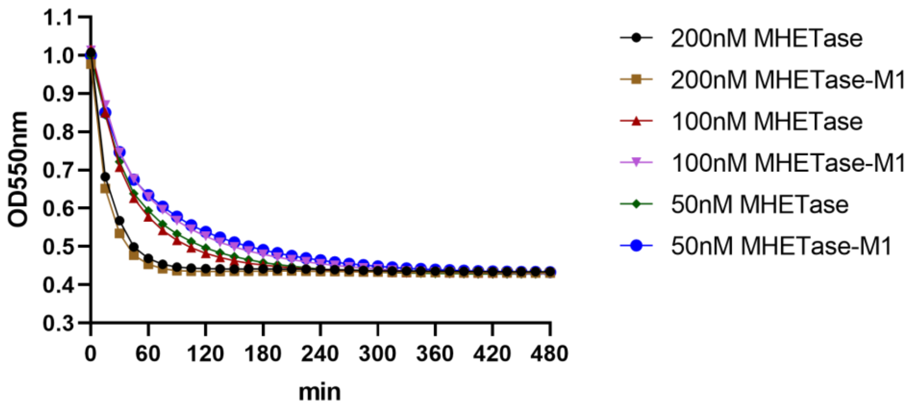

Figure S1. Colorimetric activity assay of different concentrations of MHETase at 550 nm.

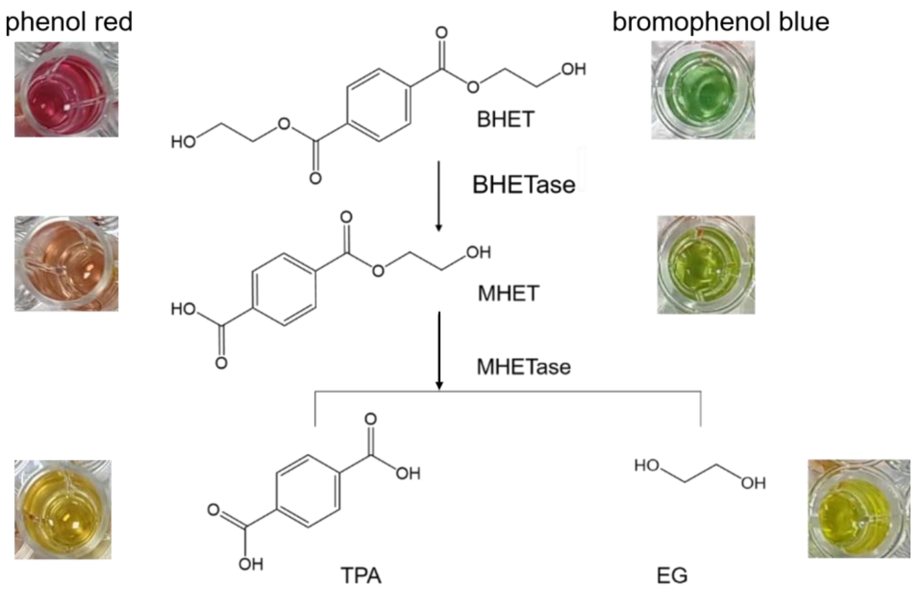

Figure S2. Schematic diagram of BHET degradation by BHETase and MHETase. The color change associated with the indicator is illustrated in the figure.
